# Striatal dopamine signals errors in prediction across different informational domains

**DOI:** 10.1126/sciadv.adq9684

**Published:** 2025-07-09

**Authors:** Kauê M. Costa, Akihiro Shimbo, Thomas Stalnaker, Nishika Raheja, Jash Mirani, Courtney Sercander, Geoffrey Schoenbaum

**Affiliations:** ^1^ National Institute on Drug Abuse Intramural Research Program, National Institutes of Health, Baltimore, MD 21224, USA.; ^2^ Department of Psychology, University of Alabama at Birmingham, Birmingham, AL 35233, USA.

## Abstract

Dopamine is classically thought to drive learning based on errors in the prediction of rewards and punishments. However, animals also learn to predict cues with no intrinsic value or biological relevance to ongoing behavior, and it is unclear whether such latent learning also relies on dopaminergic prediction errors. Here, we tested this by recording dopamine release in the nucleus accumbens and dorsomedial striatum while rats executed a sensory preconditioning task that incorporated both types of learning. We found that dopamine release in both regions correlated with errors in predicting value-neutral cues during latent learning and with errors in predicting reward during reward-based conditioning. Moreover, dopamine in the nucleus accumbens reflected inferred value in the probe test, supported by orbitofrontal cortex activity. Our findings show that dopamine signals prediction errors about both valued and neutral stimuli, consistent with its operation as a general teaching signal that supports learning across different informational domains.

## Teaser

Dopamine reflects prediction errors not only about reward but also about valueless stimuli, challenging many current theories.

## INTRODUCTION

The neuromodulator dopamine has been strongly implicated in learning about rewards and punishments (value-based learning), as across several species and tasks, dopamine activity reflects the difference between experienced and expected values or reward prediction errors (RPEs) (*1*, *2*). This has led to the powerful and influential proposal that dopamine supports something akin to a “model-free” reinforcement learning process in the brain, where learning is based only on prior learned experiences (i.e., cached values) (*1*). A critical assumption of this proposal is that dopamine signals reflect only differences in predictions about value, not the sensory properties of events (*1*). However, when navigating the world, animals also learn the relational structure of different sensorially distinct elements of the environment, even when they are seemingly irrelevant (i.e., value-neutral or latent learning) (*3*). This form of learning is linked to the construction of internal representations of the external world or a “model-based” framework, which allows for outcomes to be estimated on the fly, on the basis of the causal structure of the environment (inference).

Recent work suggests, contrary to the traditional RPE hypothesis, that dopamine may support model-based learning. Dopamine signals have been found to correlate with, and influence learning about, variables that have strictly no value, including sensory stimulus properties (*4*–*6*). Dopamine neuron activity also signals errors in predictions derived from values that must be inferred from task models (*7*–*9*). Some of this functional diversity has been related to differences in projection-specific subpopulations (*10*, *11*). For example, dopamine signaling in dorsal striatal regions, like the dorsomedial striatum (DMS), has been shown to correlate more with value-orthogonal variables than dopamine signals in the nucleus accumbens core (NAcc) (*12*–*15*). However, this heterogeneity is not sufficient to explain all the available data, because even within the NAcc, dopamine signals can correlate with nonvalue variables, like sensory salience (defined by the static properties of external stimuli) (*16*, *17*). Furthermore, individual RPE-encoding dopamine neurons in the midbrain also signal errors in the prediction of task-irrelevant sensory properties of rewards, or sensory prediction errors (SPEs) (*4*, *18*). Last, optogenetic manipulations that causally affect RPE-based learning also affect learning about the sensory features of rewards (*19*–*21*).

To reconcile these findings with classical work, several new formal hypotheses on dopamine function have been proposed. These posit that dopamine neuron activity tracks RPEs on the basis of separate predictive “threads,” “bases,” or “channels”, resulting in RPE signals that are dissociable according to their defining sensory or task-based properties (*22*–*24*). These models are essentially modifications of the dominant model-free framework to allow more complexity, diversity, or specificity in the RPE signal. However, they remain tied to learning about motivationally relevant elements with associated values. Therefore, while these updated models can explain new findings on dopaminergic errors in learning about specific reward properties and associated cues and actions, they do not predict any role for such error signaling in learning to predict events in the environment that are not related to reward or punishment.

Yet, such latent learning is common, and there is evidence that it can be influenced by brief activation and inhibition of midbrain dopamine neurons (*21*). While these results can be explained by a permissive role for phasic or even tonic dopamine in value-neutral learning (*25*, *26*), it is also possible that they are evidence that dopamine operates as an error signaling mechanism that is domain-general or multifactorial, which represents prediction errors across multiple dimensions, only one of which has a value (*4*, *16*, *18*, *27*–*31*).

This would mean that dopamine signals reflect errors in prediction across different informational domains, including domains that have no direct motivational relevance. This is a substantial departure from current hypotheses of dopamine function, as it means that a similar predictability-dependent teaching signal is conveyed through dopamine neuromodulation, and that this support most, if not all, different forms of learning. This hypothesis does not require that all errors in prediction be exactly similar, carry valence, or be bidirectional. It only requires that the responses depend fundamentally on the relational structure of elements in sensory experience (*32*, *33*). Here, we used optophysiological dopamine recordings and a classical learning theory task (*34*) to test this hypothesis by assessing whether dopamine provides an error-like signal during value-neutral latent learning. We recorded striatal dopamine signals while rats executed a three-phase sensory preconditioning task (SPC), which incorporates value-neutral, explicit value–based, and inferred value–based prediction errors in its structure (*7*, *35*).

We also took advantage of the fact that inactivating the lateral orbitofrontal cortex (lOFC) in the probe phase of this task prevents the expression of knowledge acquired during preconditioning, inactivating this region during the critical probe session while recording dopamine signals (*35*). The lOFC has been shown across different tasks to be essential for model-based inference (*35*–*37*). lOFC neurons encode the cue-cue associations that are thought to underlie the SPC effect, and inactivating the lOFC selectively disrupts behavioral responses based on inference (*35*, *38*). Therefore, by inactivating this region, we aimed to disrupt this process during the probe to see whether this would affect dopamine responses based on the inferred value.

We found that dopamine signals in both NAcc and DMS correlated with SPEs during the formation of valueless cue-cue associations. These SPE signals disappeared when a cue became well predicted by a preceding cue and returned when the cue was presented unexpectedly or when the preceding cue was swapped for another cue. In addition, in the probe session, lOFC inactivation had a selective effect on NAcc dopamine signals dependent on inference. These findings show that striatal dopamine signals reflect errors across different types of predictions, including when the value is not at issue, and that the lOFC is a critical part of this error-signaling function.

## RESULTS

**Fig. 1.**
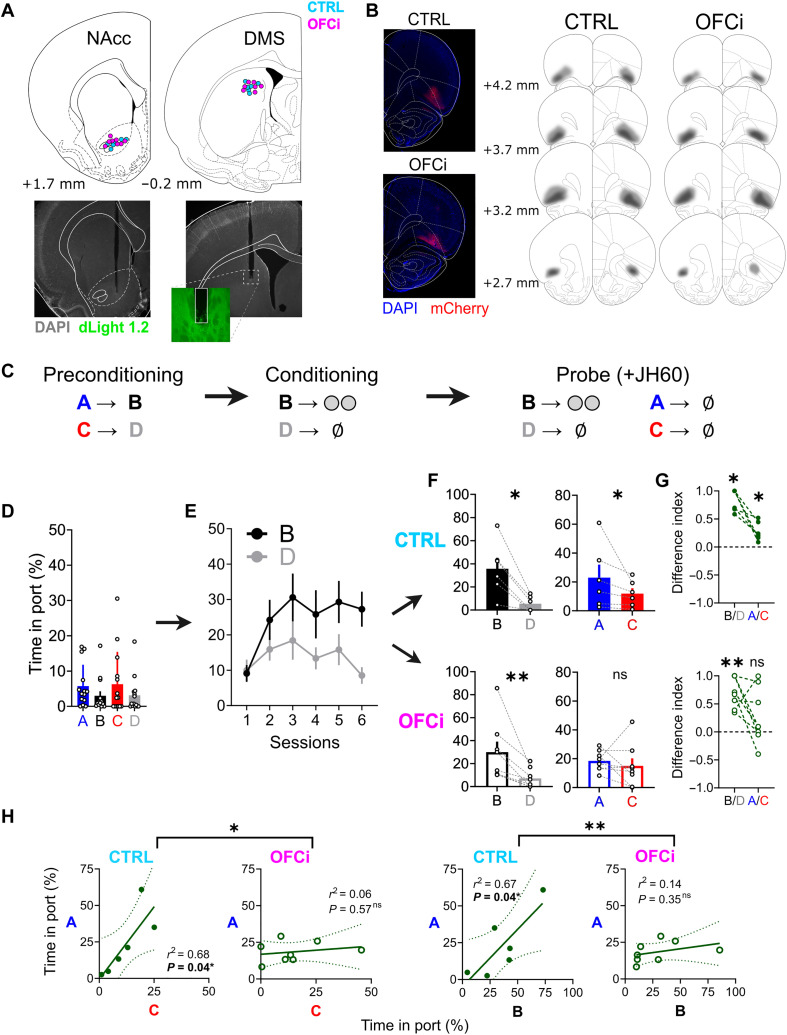
Histological verification and behavioral results. (**A**) Location of fiber tips and representative histology. (**B**) Representative microphotographs and schematic reconstructions of DREADD viral spread in both groups. Lighter shading represents the maximal spread, and darker shading represents the minimal spread. (**C**) Schematic of the behavioral task. (**D**) Behavioral response (time spent in the food port during cue) for all rats in preconditioning. Because of the absence of group effects in preconditioning and conditioning, these data were pooled for clarity (see fig. S3). (**E**) Behavioral responses in conditioning. (**F**) Behavioral responses in the probe when all rats were injected with JH60. ns, not significant. (**G**) Difference index between responses in the probe session. Positive values indicate a higher response to B or A, and negative values indicate a higher response to C or D. One-sample Wilcoxon tests against a hypothetical 0 confirmed a higher response to B versus D in both groups (*P = 0.031 for CTRL and **P = 0.008 for OFCi) and a higher response to A only in the CTRL (*P = 0.031) and not in the OFCi group (*P* = 0.148). (**H**) Linear regressions of behavioral responses in the probe. In the CTRL group, responses to A correlated with responses to B and C (A × B: *r*^2^ = 0.673, *P = 0.0454; A × C: *r*^2^ = 0.677, *P = 0.0442), as would be expected if A had been linked through inference to the value accrued to B and if responses to C were driven by a weak generalization. lOFC inactivation abolished these correlations (A × B: *r*^2^ = 0.1469, *P* = 0.3486; A × C: *r*^2^ = 0.0556, *P* = 0.5738), indicating that it suppressed the linking between cue responses at the individual level. These correlations were different between groups (A × B: *P = 0.023; A × C: **P = 0.0092), providing evidence for an effect of lOFC inactivation on inference. Data are represented as the means ± SEM. *P < 0.05; **P < 0.01.

We transfected male rats with dLight1.2 and implanted them with optic fiber cannulas in the NAcc and DMS to allow simultaneous multisite optophysiological recordings of dopamine signaling dynamics (Fig. 1A and fig. S1A) (*39*–*42*). To allow for better comparisons across rats and sessions, *z*-scored dopamine-dependent fluorescence was normalized to the maximum signal observed in each session (see Materials and Methods). We also transfected the same rats with either hM4d [inhibitory DREADD (designer receptor exclusively activated by designer drugs) receptor; *n* = 8 rats; OFCi group] or mCherry (control; *n* = 6 rats; CTRL group) in the lOFC to allow us to chemogenetically inhibit activity in this region (Fig. 1B) (*43*, *44*). After at least 4 weeks for recovery and viral expression, rats were food restricted, shaped to retrieve food pellets from the food port, and then subjected to SPC training (Fig. 1C). Dopamine signals were recorded during all sessions, and JHU37160 dihydrochloride (JH60; 0.2 mg/kg, intraperitoneally), a high-potency DREADD agonist (*45*), was injected before the probe test to inactivate the principal neurons in the lOFC of the OFCi rats, as validated previously (*43*, *44*). Against this behavioral backdrop, we analyzed dopamine dynamics in each recording site to address:

1) Whether dopamine signals in the probed locations reflected error-driven associative learning in the absence of overt value or reward.

2) Whether signals in these same sites exhibited classic RPE signals during conditioning.

3) How dopamine signals were structured during the probe test when the preconditioned cues were inferred to have values, a property known to depend on processing in the lOFC.

### Behavioral results and the role of lOFC in inference-based behavior

First, we consider the behavioral results. During the preconditioning phase, the food port response during the presentation of each cue was low and rats did not distinguish between them [Fig. 1D; two-way analysis of variance (ANOVA) for cue and group effects: *P* > 0.113 and *F* < 2.131 for all comparisons]. During conditioning, responding progressively and selectively increased to B, the cue paired with food delivery (Fig. 1E; three-way ANOVA for effects of session progression, cues, and group; session effect: *P = 0.014, *F*_5,60_ = 3.152; cue effect: **P = 0.002, *F*_1,12_ = 14.88; interaction of cue and session effects: session effect: **P = 0.008, *F*_5,60_ = 3.497; all other effects: *P* > 0.742, *F* < 0.327). Subsequently, in the probe test, all rats responded more to B, the food-paired cue, than to D, the nonreinforced cue, indicating that they had correctly learned to attribute a higher value to the food-paired cue (Fig. 1F; Wilcoxon test, *P = 0.031 for CTRL and **P = 0.008 for OFCi). Furthermore, rats in the CTRL group responded more during A than C (*P = 0.031), indicating that they had learnt the cue-cue associations in the preconditioning phase and were able to use the latent association between A and B in the probe test to infer that A (and not C) might also lead to reward (as it predicted B). Rats in the OFCi group, on the other hand, had a similar response to cues A and C (*P* = 0.313) despite showing normal B versus D discrimination, indicating a selective disruption of inference-guided behavior, replicating previous results obtained with other methods of lOFC inactivation (*35*, *46*). This effect of lOFC inactivation was also observable in the difference index (difference divided by the sum) between responding to cues B and D and cues A and C. This index was significantly above zero (one-sample Wilcoxon tests against a null hypothesis) for responses to B versus D in both groups (*P = 0.031 for CTRL and **P = 0.008 for OFCi) and for A versus C only in the CTRL group (*P = 0.031) and not in the OFCi group (*P* = 0.148). A chi-square contingency test also showed that the CTRL group has significantly more animals with an A/C discrimination index above 0.1 (at least 10% higher relative response to A) in comparison to the OFCi group (one-sided chi-square test, *P* = 0.043). Last, lOFC inactivation decoupled the response to A and B in individual subjects (Fig. 1H), somewhat similar to what was recently observed in rats exposed to cocaine self-administration (*47*), indicating that chemogenetic lOFC inactivation during the probe affected the ability of the rats to link or integrate learning in the initial and the conditioning phases to infer reward.

### Dopamine signals in the NAcc and DMS reflect predictability during preconditioning

**Fig. 2.**
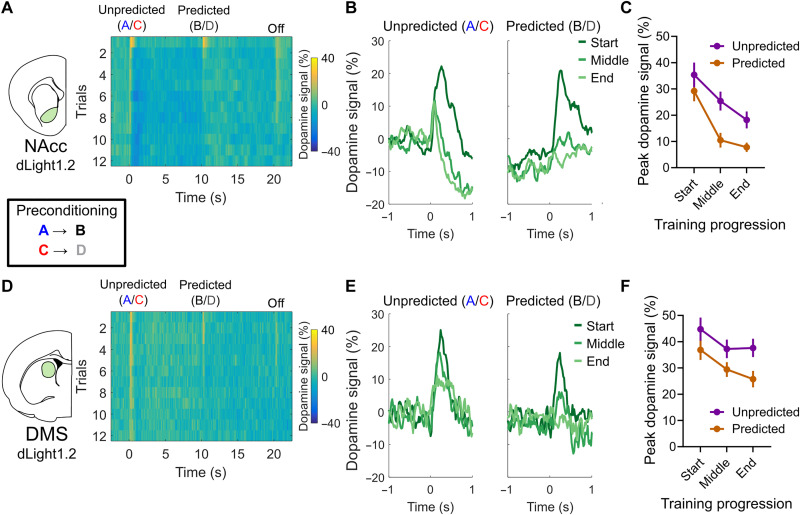
Dopamine signals in the NAcc and DMS reflect general prediction errors during sensory preconditioning. (**A**) Heatmap representation of average NAcc dopamine responses in the preconditioning phase, during all 12 preconditioning trials, recorded with dLight1.2 (*n* = 14 rats). Note the larger dopamine responses during the first trials to both the unpredicted and predicted cues and the progressive diminishment of the responses with subsequent trials, which is more pronounced for the responses to predicted stimuli. (**B**) Average traces of NAcc dopamine responses to predicted and unpredicted cues during the start (first two trials), middle (trials 6 and 7), and end (trials 11 and 12) of the preconditioning phase. (**C**) Peak NAcc dopamine responses measured over the course of the preconditioning training. Note the similar responses to both types of cues in the beginning of training and the significant difference between responses to predicted and unpredicted cues. (**D** to **F**) Similar to (A) to (C) but, for DMS dopamine signals, simultaneously recorded with NAcc signals. We highlight that in the DMS, the progressive reduction of dopamine responses to both types of cues was not as strong as in the NAcc. Data are represented as the means ± SEM. *P < 0.05; **P < 0.01.

We next asked how dopamine responses were structured during the preconditioning phase when the rats were exposed to arbitrary pairings of neutral cues in the absence of any value, overt reward, or even response requirement. The structure of these responses would allow us to formally rule out competing explanations for the type of information being encoded in the dopamine responses (fig. S2). Focusing on signals around cue presentation, and starting with responses in the NAcc, we observed clear dopamine peaks at the onset of each cue (A, B, C, and D). Initially, these peaks were similar (Fig. 2, A and B) and each declined with repeated exposure (two-way ANOVA for the effect of trial progression and cue predictability; trial effect: ***P < 0.0001, *F*_2,26_ = 22.34), a pattern that by itself would be consistent with just the signaling of surprise or novelty (*48*–*50*) and which has been associated with learning that underlies latent inhibition (*17*, *51*).

However, concurrently with this expected effect, we also found that peak responses to the predicted events B and D declined faster and to a greater extent than those to the unpredicted stimuli A and C (Fig. 2, B and C; cue predictability effect: **P = 0.0006, *F*_1,13_ = 20.14). This additional effect indicates that the predictive structure influences the dopamine responses to each cue, essentially providing an error-like correlate during valueless sensory-sensory learning—an SPE riding on top of novelty-related changes (Fig. 2 and fig. S2).

We observed a qualitatively similar response pattern in the DMS, where dopamine peak responses were larger at the first presentation of each cue, reduced over repeated exposures, and were lower for predicted events than for unpredicted events (Fig. 2, D and E; trial effect: *P = 0.023, *F*_2,54_ = 4.039; cue predictability effect: **P = 0.004, *F*_1,27_ = 9.577). However, the changes in peak responses over time were less pronounced and followed at a slower progression rate than in the NAcc. A multivariate ANOVA accounting for region differences showed a main effect of trial progression (*P* < 0.0001, *F*_2,52_ = 21.02), predictability (predicted versus unpredicted: *P* = 0.0002, *F*_1,26_ = 25.50), and the region of recording (NAcc versus DMS: *P* < 0.0001, *F*_1,26_ = 19.21), as well as a significant interaction effect between region and trial progression (*P* = 0.0271, *F*_2,52_ = 3.871) on peak dopamine signals during the preconditioning phase. This indicates that perhaps the changes observed in DMS responses were following those in the NAcc in time, consistent with previous evidence suggesting an ascending ventromedial to dorsolateral hierarchy of learning-induced plasticity in midbrain-striatal circuits (*52*, *53*).

**Fig. 3.**
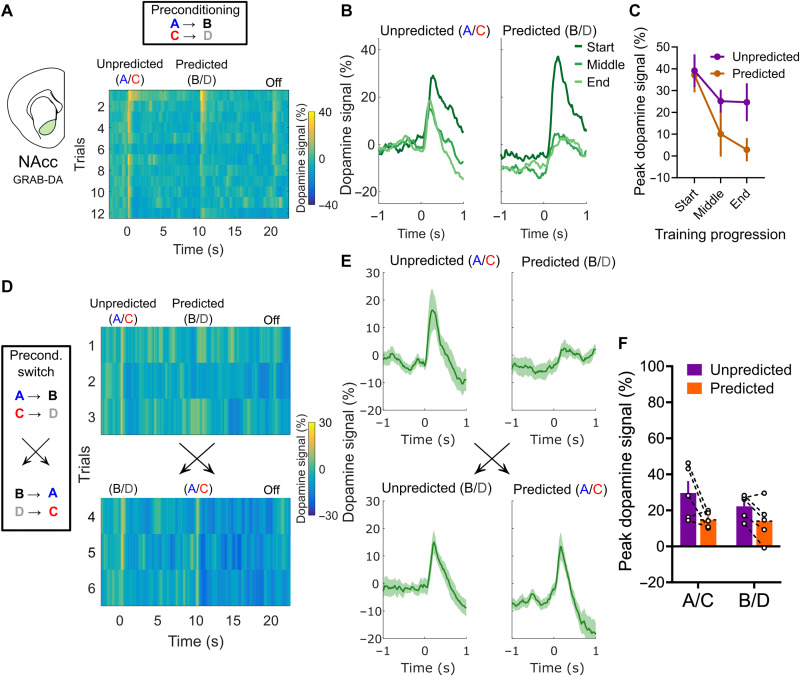
Dopamine encoding of sensory-sensory prediction errors in the NAcc depends on the predictive relationship between cues. (**A**) Heatmap representation of the average NAcc dopamine signals during all trials of preconditioning training in a separate cohort of rats (*n* = 5 rats), recorded with GRAB-DA2m. Note that the response pattern is similar to those recorded with dLight1.2. (**B**) Average traces of dopamine responses during the start, middle, and end of preconditioning, defined the same way as in Fig. 2. (**C**) Peak dopamine responses during preconditioning training in this cohort of rats. Note the similar pattern to the cohort in Fig. 2. (**D**) Heatmap representation of dopamine signals in the preconditioning switch session. In the first three trials, the structure of the task was the same as in the previous training sessions, with A preceding B and C preceding D. In the last three trials, that order was switched, with B coming before A and D before C, with no external signaling to the rats. Note that responses to B and D, which were almost entirely suppressed in trial 3, were immediately restored in trial 4 when these cues presented unexpectedly, and responses to cues A and C, which were unexpected, start to become slightly more suppressed once they become predicted by cues B and D. (**E**) Average dopamine responses to each type of cue before and after the switch. (**F**) Peak dopamine responses to cues A/C and B/D when they were both predicted (first three trials for B/D and last three trials for A/C) and unpredicted (first three trials for A/C and last three trials for B/D). For both sets of cues, regardless of their identity or exposure history, NAcc dopamine responses were larger when they were unpredicted than when they were predicted. Data are represented as the means ± SEM.

To confirm the importance of the predictive structure in determining the observed changes in dopamine response rather than the initial novelty of the cues or habituation to their presentation, we trained a second group of rats (*n* = 5), this time transfected with GRAB-DA2m and implanted with an optic fiber cannula only in the NAcc and without a viral injection of DREADDs into the lOFC. These rats underwent the same preconditioning training as described before (Fig. 3, A to C), except that we added a session at the end in which we switched the order in which the cues were presented (Fig. 3D). In other words, in this added session, the rats initially received presentations of A → B and C → D, as they had the prior two sessions, followed by presentations of B → A and D → C. If the changes in dopamine signals were due to the predictive structure of the cues, then the response should be immediately restored to D and B when they appear unpredicted by another cue, and it should slowly diminish to A and C, as the new predictive structure is learnt. Consistent with this idea, we first found that NAcc dopamine signals in this new group showed a similar SPE signature as in the previous dLight1.2-transfected rats during the first two sessions—responses to predicted cues B and D were lower than responses to the unpredicted cues A and C—confirming that this finding is robust across experimental groups and dopamine sensors (Fig. 3, A to C; trial effect: **P = 0.0027, *F*_2,8_ = 13.57; cue predictability effect: *P = 0.045, *F*_1,4_ = 8.25). Then, when the order of cue presentation was reversed in the second half of the added preconditioning session, the responses to B and D, now unpredicted, were restored, and responses to cues A and C, now predicted, were slightly reduced (Fig. 3, D to F; two-way ANOVA for effect of cue predictability—unpredicted versus predicted—and cue identity—B/D versus A/C; predictability effect: *P = 0.0178, *F*_1,4_ = 15.06; cue effect: *P = 0.0428, *F*_1,4_ = 8.593; interaction effect: cue effect: *P* = 0.4458, *F*_1,4_ = 0.7136). This pattern strongly suggests that NAcc dopamine reflects the predictive structure of cue presentation in the same way that the classic RPE response reflects the order of presentation of a cue and an event with an overt value—an SPE for the lack of a better term.

**Fig. 4.**
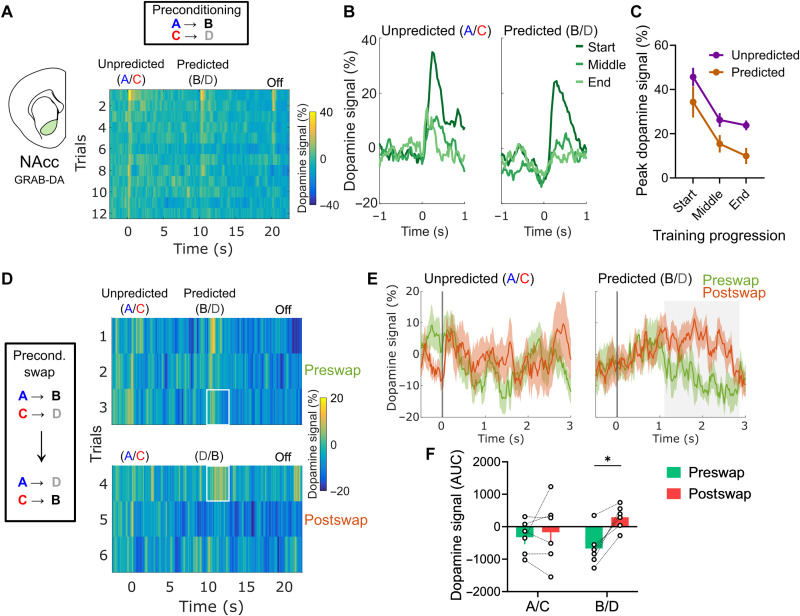
Sensory prediction error–like dopamine responses in the NAcc reflect specific stimulus-stimulus associations. (**A**) Heatmap average NAcc dopamine responses during preconditioning training in a new cohort of rats (*n* = 6) recorded with GRAB-DA2m. The response pattern is similar to those recorded with dLight1.2 and to those observed with GRAB-DA2m in the previous experiment (Fig. 3). (**B**) Average traces of dopamine responses during the start, middle, and end of preconditioning, as in Figs. 2 and 3. (**C**) Peak dopamine responses during preconditioning training. Note the similar pattern to the cohort in Fig. 2. (**D**) Heatmap of dopamine signals in the preconditioning swap session. In the first three trials, the structure of the task was the same as in the previous training sessions, with A preceding B and C preceding D. In the last three trials, the predicted cues B and D were swapped, with B coming after C and D after A, with no external signaling to the rats. Note that responses to B and D, which were again substantially suppressed in trial 3, were restored in trial 4 when the swapping of these cues violated their previous associations with cues A and C. (**E**) Average dopamine responses to each type of cue before and after the B/D swap. The difference in dopamine responses observed after the swap developed slowly after cue onset. (**F**) AUC of dopamine responses to cues A/C and B/D before and after the swap. The AUC was calculated on the time window signaled in gray in (E). Note that the AUC values to cues A and C do not change significantly after the swap, but AUC values of responses to cues B and D, which were previously negative, became uniformly and significantly more positive. Data are represented as the means ± SEM. *P < 0.05.

Last, to demonstrate that the observed changes in dopamine response reflect the specific cue-cue associations learnt during preconditioning rather than nonspecific relationships (e.g., B and D are always preceded by another stimulus, but its identity does not matter), we trained a third group (*n* = 6 rats), also transfected with GRAB-DA2m and implanted with an optic fiber cannula only in the NAcc, with no DREADDs in the lOFC. These rats received the same preconditioning training used in the previous experiments (Fig. 4, A to C), but now, in the end of the third session, we swapped the identity of the predicted cues (Fig. 4D). This means that the rats again initially received presentations of A → B and C → D, as they had in the prior two sessions, but this was then followed by presentations of A → D and C → B. This design ensured that any differential response to cues B and D after the swap could not be attributed to a generalized sensory expectation or adaptation but instead must be related to the specific sensory identity of the predicted cues.

The results in this new cohort again replicated the dopamine response pattern during preconditioning observed in both previous groups of rats (Fig. 4, A to C; trial effect: **P = 0.0017, *F*_2,10_ = 12.89; cue predictability effect: *P = 0.0313, *F*_1,5_ = 8.793). Then, when the initial cues in each pair were unexpectedly swapped, there was an increased response to the predicted cues (Fig. 4, D to F). Specifically, there was a larger dopamine response in this trial compared to the responses in the last trial of exposure to the cues in their previously learned pairs. This response occurred somewhat later compared to the responses evoked by cue B or D in the other experiments in this study; instead of reaching its peak within a second after cue onset, the dopamine response to the swapped cues developed over the course of multiple seconds, being observable up to 3 s after cue onset. We would speculate that this slower time course reflects the processing time required to disambiguate the new cue from what was expected. To quantify this slower response, we measured the area under the curve (AUC) within 1 to 3 s after cue onset. This analysis confirmed that swapping the identity of the predicted cues changed the dopamine responses (Fig. 4F; two-way ANOVA; pre- versus postswap effect: **P = 0.0081, *F*_1,5_ = 18.03), specifically by changing the response to the predicted cues B and D (Sidak’s post hoc test, *P = 0.0469). This result indicates that the dopamine responses to changes in predictive contingencies between neutral cues reflect identity-specific cue-cue associations.

### Dopamine signals in the NAcc reflect classical RPEs during conditioning

**Fig. 5.**
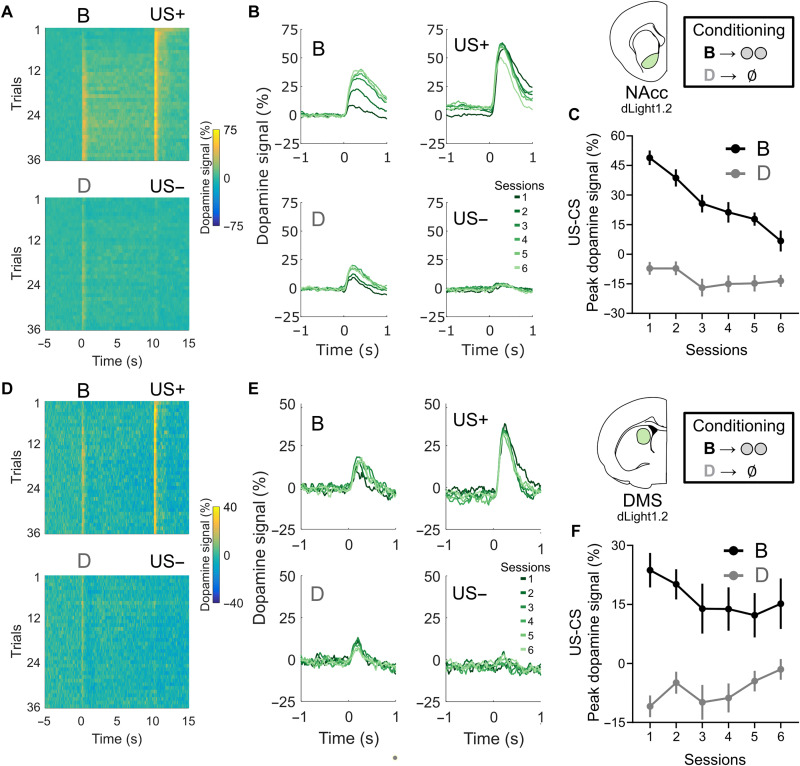
Dopamine signals in the NAcc and DMS reflect standard, value-based, “reward” prediction errors during classical conditioning. (**A**) Heatmap representation of average NAcc dopamine signals in all trials of the conditioning phase of the cohort recorded with dLight1.2 (*n* = 14 rats). Note the progressive reduction of responses to US presentations and the concurrent increase of responses to cue B, the reinforced CS+, with less changes in the responses to cue D, the unreinforced CS−. (**B**) Average traces of dopamine signals for each session (six trials per condition per session) of conditioning. (**C**) Difference between the peak NAcc dopamine responses to US and CS presentation for both cues B and D. A progressive decrease in this difference is a key indicator of an RPE response (*7*). (**D** to **F**) Same as (A) to (C) but for DMS dopamine signals. Note that DMS dopamine also shows signs of RPE encoding but much less prominently as the NAcc. Data are represented as the means ± SEM. *P < 0.05; **P < 0.01.

Consistent with numerous prior studies, we found that peaks in dopamine signals in the NAcc strongly signaled RPEs during the conditioning phase of the task (Fig. 2) (*12*, *14*). Peaks in signals were initially high in response to pellet delivery and then decreased over time, while peaks in response to the onset of the pellet-paired cue B increased (Fig. 5A). This pattern could be resolved on a trial-by-trial (Fig. 5A) or session-by-session basis (Fig. 5B), with the difference between peak responses to the pellet delivery (US) and the cue (CS) becoming progressively lower with training (Fig. 5C; two-way ANOVA for effects of session progression and cue—B versus D; session effect: ***P < 0.0001, *F*_5,65_ = 14.48; cue effect: ***P < 0.0001, *F*_1,13_ = 153.2; interaction effect: ***P < 0.0001, *F*_5,65_ = 9.809) (*7*). Responses to the nonreinforced cue D did not change significantly over time. These results confirm that dopamine signals in the NAcc—recorded in the current rats and fiber locations and using this task and these methods—exhibited classic RPE correlates during conditioning.

Furthermore, we found that dopamine peaks in the DMS also seemed to reflect RPEs, albeit again with a weaker effect size and with a slower update rate as compared to the NAcc (Fig. 5, D to F; session effect: *P* = 0.1490, *F*_5,65_ = 0.892; cue effect: ***P < 0.0001, *F*_1,13_ = 35.89; interaction effect: **P = 0.0086, *F*_5,65_ = 3.402). This RPE effect was much clearer in the analysis of the AUC of the dopamine signals right after cue presentation (fig. S6B, right panel; session effect: *P* = 0.492, *F*_5,65_ = 0.892; cue effect: ***P < 0.0001, *F*_1,13_ = 36.26; interaction effect: *P = 0.0221, *F*_5,65_ = 2.842), where post hoc Sidak tests showed a significant difference between DMS dopamine responses to cue B on session 1 and responses on sessions 3 (*P = 0.0057), 4 (*P = 0.0295), and 5 (**P = 0.007). The presence of an RPE signal is further supported by a *t* test comparison between the peak dopamine response in the first session and the average response of the last two sessions (*P = 0.049). While the RPE-like effects were weaker relative to those observed in the NAcc, this is to be expected given the current literature (*12*, *13*), and the general response pattern parallels our findings in the preconditioning phase, where changes to signals in the DMS followed a slower trajectory over sessions than those in the NAcc.

### NAcc dopamine responses to cues with an inferred value depend on lOFC activity

Last, we arrive at the probe phase of the main SPC experiment. In the previous sections of this study, we have collapsed the two groups because there was no effect of group on any of the previous comparisons (figs. S3 and S4; three-way ANOVA for effects of session/trial progression, cues, and group; all group main or interaction effects: *P* > 0.1, *F* < 0.5). This was done to provide more clarity and simplicity to the reader and to avoid cluttering of the graphs (data for the preconditioning and conditioning sessions divided by lOFC inactivation groups can be seen in figs. S3 and S4). For the probe test, as we are interested in the effects of lOFC inactivation, we report the data divided by the OFCi and CTRL groups. Furthermore, the data presented in the main manuscript are restricted to rats that showed strong discriminative conditioning, as this is necessary for revealing preconditioning effects (*54*, *55*).

**Fig. 6.**
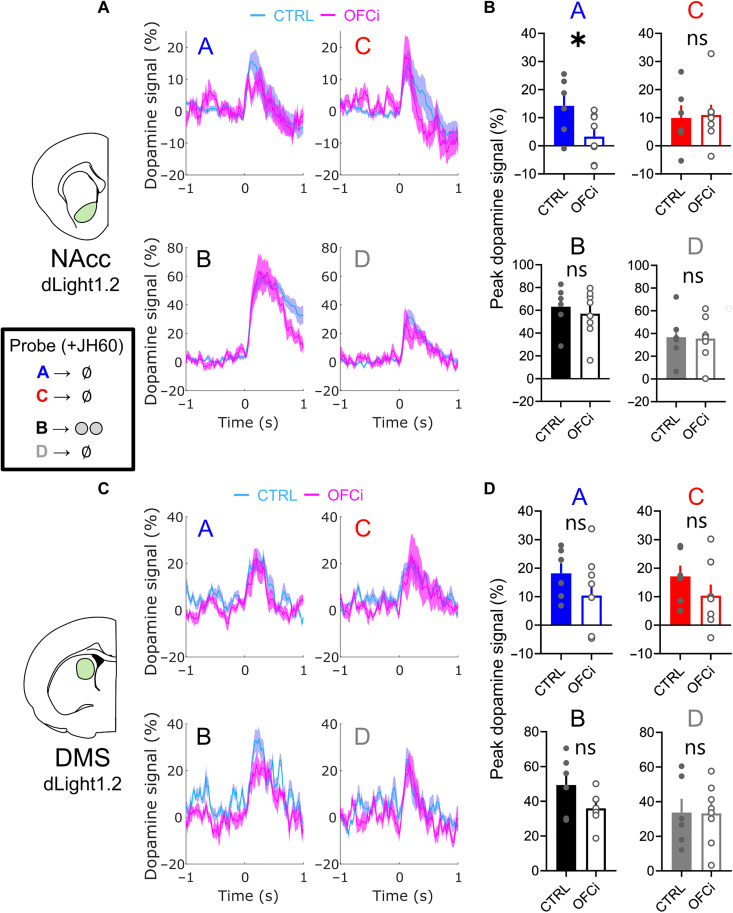
Dopamine signals in the NAcc reveal a contribution of lOFC to learning-dependent dopamine signals during probe. (**A**) Average traces of NAcc dopamine responses to all cues in the probe test for both CTRL (*n* = 6 rats) and OFCi (*n* = 8 rats) groups. All recordings and behavior in this session were performed after the intraperitoneal injection of JH60 (0.2 mg/kg), which inhibited lOFC activity in the OFCi group. Note that the only difference between responses in the two groups is a blunted dopamine peak to A in the OFCi group. (**B**) Peak NAcc dopamine responses to all cues in the probe session, confirming the significant difference only in responses to A between CTRL and OFCi rats. (**C** and **D**) Same as in (A) and (B) but for DMS dopamine responses. In the DMS, there was no significant difference between responses to any of the cues between the CTRL and OFCi rats. Data are represented as the means ± SEM. *P < 0.05.

We found that in the NAcc, responses to the reinforced cue B were significantly higher than to the unreinforced cue D in both the CTRL and OFCi groups (Fig. 6, A and B; paired *t* tests; CTRL: **P = 0.002; OFCi: **P = 0.007). In the DMS, there was no significant difference between responses to B and D cues in either group (Fig. 6, A and B; paired *t* tests; CTRL: *P* = 0.087; OFCi: *P* = 0.466), which is likely reflective of the less pronounced RPE encoding observed in the DMS compared to the NAcc and the lower number of trials in the probe session. In the CTRL group, dopamine responses to cues A and C were similar during the probe phase (Fig. 6). This was true in both areas (paired *t* test, A versus C in the CTRL group; NAcc: *P* = 0.326; DMS: *P* = 0.801). The fact that measured dopamine signals did not differ for the two cues was unexpected, in light of the differential behavior of these rats to the two cues in the probe test (Fig. 1) as well as prior unit recording data showing that dopamine neuron spiking is higher to cue A than to cue C (*7*). These results are reminiscent of prior demonstrations that striatal dopamine release and dopamine neuron firing are not always correlated and that certain behavioral variables can be observed in one measure but not the other (*56*).

Nevertheless, even if the neurotransmitter signals appear similar, the hypothesized circuit function predicts that signals should reflect different information for the two cues; specifically, while responses to cue C might reflect nonspecific generalization and perhaps elevated salience because of the long period since it was last seen, responses to cue A should reflect, at least in part, the specific predictive relationship between A and B and between B and reward. We know from prior work in this setting that this is the case for the behavior to A, as it is sensitive to devaluation of the predicted reward (*57*). Critically, behavior based on this information is selectively sensitive to inactivation of lOFC (*35*). Thus, inactivation of lOFC should selectively affect dopamine responses to A. Consistent with this prediction, we found that lOFC inactivation did reduce the relative peak responses to cue A in the NAcc (Fig. 6, A and B), with responses to all other cues being relatively similar (paired *t* tests; A versus C in the OFCi group; NAcc: *P = 0.044) (unpaired *t* tests; A response in CTRL versus OFCi; NAcc: *P = 0.043; all other comparisons of CTRL versus OFCi showed *P* > 0.05 for both the NAcc and DMS). Furthermore, a two-way ANOVA considering the responses to A and C across experimental groups revealed a significant interaction effect between cues and group (*P = 0.0335, *F*_1,12_ = 5.764), and post hoc multiple comparison Fisher least significant difference tests also confirmed differences in response to A between groups (*P = 0.0491) and to A versus C in the OFCi group (*P = 0.0365). A similar analysis for the responses to cues B and D only found a significant effect of cue (****P < 0.0001, *F*_1,12_ = 38.33), and post hoc tests showed significant differences in responses to B and D in both CTRL (***P = 0.0007) and OFCi (**P = 0.0011) groups, with no significant differences between groups, confirming the selectivity of lOFC inactivation on the responses to cue A. The major findings reported here are reproduced when analyzing the AUC of the dopamine signals (fig. S6).

Last, we reasoned that if the SPE correlates in the preconditioning session are driving the latent cue-cue associative learning that determines the A/C discrimination in the probe, then it is the degree to which responses to the predicted cues were suppressed during preconditioning that should predict the magnitude of the sensory preconditioning effect observed in the CTRL group in the probe session. Likewise, this relationship should be abolished by lOFC inactivation. To test this, we measured the correlation between the ratio of NAcc dopamine peak responses to cues between the early and late phases of preconditioning (i.e., the degree to which a given cue response was suppressed by preconditioning training) and the A/C difference index in the probe (i.e., a normalized measure of the behavioral sensory preconditioning effect). We found that the suppression of dopamine responses to predicted cues (B and D) in the CTRL group was inversely correlated with the magnitude of A/C discrimination during the probe (fig. S7). That means that the more the responses to predicted cues were suppressed during preconditioning (the main signature of SPE signaling), the better the expression of cue-cue learning in the probe. This is exactly what would be expected if the NAcc dopamine SPE signaling was driving the latent valueless sensory-sensory associative learning. This relationship is not observed in the OFCi group, further demonstrating the effect of lOFC inactivation to disrupt cue-cue associations.

## DISCUSSION

Here, we recorded dopamine in a three-phase SPC task and found that dopamine responses in both the NAcc and DMS reflected errors in predicting neutral cues during the initial preconditioning phase of training. These signals, detected at sites that also showed classic RPE signals during subsequent conditioning, were dissociable from sensory salience and novelty. Thus, the results replicate previous findings linking striatal dopamine responses to the salience and signaling of value-based prediction errors (*1*, *12*, *16*, *17*, *49*) while also showing a previously undescribed value-independent error–like response during learning about neutral cue pairs. The importance of the latter finding is bolstered by previous work using optogenetics showing that dopamine activity is both necessary and sufficient for driving sensory-sensory learning in a similar preconditioning task (*21*) and previous work showing that systemic blockade of dopamine receptors disrupts preconditioning learning (*58*). These causal manipulations could not formally distinguish between an error-driven signal and a more general permissive role of dopamine for value-neutral learning or simply the effects of attentional signals unrelated to the relational structure of cue presentation (fig. S2). Thus, the current data are critical in ruling out these possibilities in favor of a prediction error–like mechanism.

Showing error signals in this setting goes beyond prior work by us and others that showed error signals during learning of specific features of otherwise valuable events (i.e., rewards) (*4*, *8*, *18*, *19*, *59*), which can be explained with adjustments to model-free learning algorithms, such as the addition of dissociable “threads,” “bases,” or “channels” for keeping track of different components of rewarding events (*22*–*24*). These models cannot easily explain why neutral cues evoke error signals. Instead, the response patterns observed here to neutral cues are much better explained by a model-based process or at least a hybrid model incorporating something like successor representations (*27*, *28*, *30*, *44*). It also adds to previous work suggesting the encoding of persistent SPEs by dopamine signals in the tail of the striatum (*12*), as we demonstrate the existence of dopamine SPE signaling in the same regions where RPEs are observed (unlike in the tail of the striatum) and in a task paradigm that links these SPEs to sensory-sensory learning.

The results reported here, coupled with much prior work (*1*, *4*, *8*, *14*, *18*, *19*, *31*, *59*), suggest that dopamine activity in each target region is shaped by multiple factors or dimensions that characterize events, of which the value is but one component (*27*, *28*, *30*, *44*). This broader interpretation holds better explanatory power than several alternatives. For example, one potential challenge to our conclusion that dopamine transients in the NAcc and DMS reflect more than a value is the expansion of the concept of “value” itself. For example, novel sensory cues may be assigned an intrinsic value, given that they could potentially convey information about the external world (*60*–*62*), or perhaps previous experiences, in the home cage or during shaping, led the rats to acquire the general belief that sounds are broadly predictive of rewards before preconditioning (*63*). These explanations seem unlikely, as preconditioned cues trained in this manner do not support conditioned reinforcement (*64*). Furthermore, if these cues were intrinsically valuable, like a primary reward, then the dopamine responses to the unpredicted cues A and C should have increased over time during preconditioning, just like responses to B during conditioning, but they did not. Instead, the observed pattern, in which the measured dopamine responses to predicted cues were inhibited faster, seems more compatible with the formation of a predictive association between two cues without an intrinsic value.

A related set of ideas is that dopamine responses, especially in the NAcc, track overall stimulus salience (*16*, *29*, *49*). This component of the signal differs from an isolated value prediction error because it factors in both intrinsic attributes of the cues themselves and the relational structure of task elements, such as novelty, stimulus intensity, and unexpectedness. Its presence should increase associability or learning about a cue, both at that time and subsequently. Our findings here and in prior causal work are generally compatible with this interpretation, as predicted cues would, by definition, be less unexpected than unpredicted cues. However, we reiterate that this broad definition of salience is also fundamentally dependent on errors in predicting events. Novelty, sensory salience, and signaled sensory and value-based prediction errors could all be conceptualized as prediction errors that operate on different variables and across different hierarchical structures of timescale and information content (*4*, *30*). Similar frameworks have been proposed to shape perception and general cognition, and the idea that the brain itself is fundamentally dedicated to processing, and minimizing, prediction errors is currently an influential and powerful hypothesis (*65*).

A demonstration that dopamine is critical to learning about nonrewarding events carries potential implications for its role in diseases, especially the dopamine hypotheses of schizophrenia and drug addiction. It has been proposed that dopamine hyperactivity may attribute rewarding properties to irrelevant stimuli, promoting delusions (*66*). Our results suggest that this aberrant associativity may extend beyond reward attribution and affect associative learning and credit assignment across all forms of experience, which resonates with recent proposals arguing for a general dysfunction of prediction error processing in schizophrenia and psychosis (*67*). Likewise, one of the main hypotheses on drug addiction is that drug abuse drives dopamine release to create an aberrant reward signal (*68*). Our results suggest that drug-induced dopamine release, especially in the NAcc, may affect other forms of learning beyond reward attribution, which may compound with the known rewarding properties of addictive drugs. This is congruent with the finding that drug use not only changes behaviors related to reward but also affects decision-making across several different tasks, especially those that rely on model-based learning (*69*).

The involvement of dopamine in model-based learning is also supported by the effects of lOFC inactivation in the probe test, where it disrupted behavioral response and dopamine signaling in the NAcc to cue A, the cue endowed with an inferred value by virtue of the prior training. This result is consistent with the impact of muscimol inactivation of lOFC on response in the probe test in our prior work (*35*) and with the widespread observation that the lOFC is strongly associated with inferential or model-based behavior across a variety of tasks, modalities, and species (*37*, *70*–*72*).

Dopamine recordings in the probe test did show some unexpected features. In the control group, the dopamine response was similar to both cue A, the preconditioned cue predictive of reward, and cue C, the control cue. If the only mechanism at work here was the one based on inference and if the only information represented by the error signal was a value, then the signal should preferentially have shown up to cue A. In this regard, the results contrast with the results of a previous single unit recording study (*7*), in which the firing of putative dopamine neurons in the ventral midbrain differed between cues A and C in the probe test of a similar task.

However, there may be a number of non–mutually exclusive explanations for this finding. For starters, the optophysiological methods used here may be not sensitive enough to pick up what may be a very subtle difference in the dopamine responses to cues A and C in the control group, or to put it another way, other components of the dopamine response (i.e., local features of the spatiotemporal patterns of release, differential release from individual axon terminals, etc.) may better reflect inferred prediction errors than bulk responses to cue onset. As a conjecture, it is also possible that in the unit recording study (*7*), the sampled neuronal population may have contained a disproportionate number of neurons that projected to other striatal regions not sampled in our current work, where different variables may be represented. This is important, as recent work has suggested that it is critical to match genetic- and projection-specific dopamine subpopulations to their release site to properly relate the effects of somatic firing on dopamine release (*73*, *74*). However, the methods and analyses applied here, which fit the standard of the field, were successful in detecting prediction errors in preconditioning and conditioning, including classically defined RPEs, as well as some differences imposed by lOFC inactivation. Therefore, this result seems unlikely to be due to these artifactual causes.

More likely, dopamine release may follow a different time course than spiking activity, with different sensitivities to aspects of our design. Such speculation is consistent with recent work showing that dopamine neuron firing and release in target regions can be sometimes dissociated, likely due to the sculpting effects of local striatal mechanisms (*56*, *75*–*77*). Similar responses in controls to the two preconditioned cues might also reflect a greater sensitivity of these signal dynamics to commonalities across the cues that are unique to our task. For instance, in preconditioning, all the cues are initially presented without any reward, and this similarity may be the foremost determinant of bulk dopamine signals in the probe, even if other processes are in play. This might be quite different for other tasks designed to study inferential behavior, like reinforcer devaluation, where no common unrewarded latent state can be inferred during the initial learning phase (*8*). Within this framework, one might predict that it would require a selective manipulation of areas that process inferential information—such as the lOFC (*44*)—to reveal a potential difference in dopamine responses that depend on such processes, which is what we found.

Our findings that llOFC inactivation selectively disrupts dopamine responses related to inferential processes are in line with previous work on the role of the llOFC in sensory preconditioning (*35*, *38*). Furthermore, we found that lOFC inactivation disrupts the correlation between the behavioral responses to different cues in the probe session and between dopamine SPE signaling in preconditioning and the SPC effect in the probe, which suggests that lOFC inactivation leads to a reorganization of the internal associative representation, or cognitive map, of the task (*37*, *44*, *70*). It is worth noting that disruptions of behavioral response correlations in SPC are also observed following chronic drug exposure (*47*), a procedure that also affects behavioral dopamine signals (*78*), including to neutral cues (*79*), which indicates that abnormal association-related OFC signals to the midbrain dopamine system may play an important role in the behavioral dysfunction seen in drug addiction.

The connection between lOFC function and dopamine behavioral signals has been well established at the anatomical, physiological, and behavioral levels across different species. For starters, the lOFC is the cortical region with the densest and most abundant direct projections to VTA dopamine neurons (*80*). Functional work using juxtacellular recordings of dopamine neurons has demonstrated that stimulation of lOFC can both excite and inhibit VTA dopamine neuron activity (*81*, *82*), indicating that excitatory lOFC neurons likely project both directly to VTA dopamine neurons and to inhibitory neurons that synapse onto dopamine neurons. This suggests that the lOFC can bidirectionally control dopamine neuron activity, although it is not known whether the excitatory and inhibitory effects of lOFC stimulation are mediated by different subpopulations of lOFC neurons or whether different subtypes of dopamine neurons are differentially controlled by the lOFC.

Several behavioral neurophysiological studies have demonstrated that inactivation or lesion of the lOFC impairs the encoding of task-related information by VTA dopamine neurons, especially information related to reward expectation, task structure, inference, and value-orthogonal information like identity prediction errors (*81*, *83*–*86*). The lOFC also projects directly to the NAcc (*87*, *88*), so lOFC neurons could potentially inhibit dopamine neurons by exciting direct pathway striatal projection neurons or even form axo-axonal synapses with dopamine axons and directly drive dopamine release, but to our knowledge, these have not been demonstrated and are only speculative possibilities. Last, the lOFC projects to several other regions that are known to be involved in regulating dopamine neuron activity, such as the amygdala, hypothalamus, and other cortical regions (*89*). In summary, there are multiple circuit pathways by which lOFC neurons can excite dopamine neurons, including direct projections to these cells, and it has been well described that lOFC inactivation and lesions have selective effects on task-related dopamine signaling, all of which is compatible with the observed effects of lOFC inactivation in this study. Future work should aim to precisely elucidate how specific lOFC circuits can affect different dopamine neuron subtypes during behavior.

In conclusion, our findings support the proposal that dopamine signaling in the striatum mimics a general, multifactorial prediction error term that comprises different variables and whose specific information content varies across different regions. These prediction errors in the NAcc and DMS reflect the relational structure of value-neutral sensory-sensory associations, as well as cached value-based associations and novelty, which means that dopamine release in different regions reflects predictability across different types of experience and information domains. We also present evidence that accumbal dopamine responses to cues with an inferred value are differentially sensitive to lOFC disruption, suggesting that dopamine signals that appear similar may be dependent on different underlying mechanisms according to their task relevance.

## MATERIALS AND METHODS

### Experimental model and subject details

Experiments were performed on a total of 31 male Long-Evans rats (>3 months of age at the start of the experiment, Charles River Laboratories) housed on a 12-hour light/dark cycle at 25°C. Rats were food restricted to ~85% of their original weight for the duration of the experiments and were tested at the National Institute on Drug Abuse Intramural Research Program in accordance with National Institutes of Health (NIH) guidelines determined by the Animal Care and Use Committee, which approved all procedures. All rats had ad libitum access to water during the experiment and were fed 16 to 20 g of food per day, including rat chow and pellets consumed during the behavioral task. Behavior was performed during the light phase of the light/dark schedule. Before surgery and each experiment, rats were handled by the experimenter, as previously described (*43*, *44*).

### Surgical procedures

For the main preconditioning experiment, rats (*n* = 24) were anesthetized with 1 to 2% isoflurane and prepared for aseptic surgery. They received unilateral infusions of AAV5-hSyn-dLight1.2 into the NAcc [anteroposterior (AP) +1.7 mm, mediolateral (ML) +1.7 or −1.7 mm, and dorsoventral (DV) −6.3 and −6.2 mm from the brain surface], DMS (AP −0.4 mm, ML +2.6 or −2.6 mm, and DV −3.7 and −3.6 mm from the brain surface), and dorsolateral striatum (AP +0.2 mm, ML +3.8 or −3.8 mm, and DV −3.7 and −3.6 mm from the brain surface). Recordings from the dorsolateral striatum were poorly correlated with our task events and seemingly correlated more with individual movements; as that was not the focus of our study, these signals were not further included in our analysis. Separate groups of rats received unilateral infusions of AAV9-hSyn-GRABDA2m (*n* = 5 rats for the “switch” experiment and *n* = 9 rats for the “swap” experiment; of the latter nine rats, one had a misplaced infusion and two were excluded because of a malfunction in one of the operant boxes, leaving a final *n* = 6 for the “swap” experiment) or AAV9-hSyn- GRAB-DA-mut (*n* = 2 rats) only in the NAcc and no additional viral infusions. A total of 0.7 μl of each dopamine sensor–transfecting virus was delivered in each site at 0.1 μl/min via an infusion pump. Optic fiber cannulas (200-μm diameter; Neurophotometrics, CA) were implanted in each site in the location of the second (most dorsal) viral infusion.

Rats for the main preconditioning experiment, which were transfected with dLight1.2 and implanted in different areas of the striatum, also received in the same surgery infusions of either AAV8-CaMKIIa-hM4d-mCherry (an inhibitory, G_i_-coupled DREADD; OFCi group, *n* = 12 rats) or AAV8-hSyn-mCherry (CTRL group, *n* = 12 rats), bilaterally into the lOFC (AP −3.0 mm, ML ±3.2 mm, and DV −4.4 and −4.5 mm from the brain surface) (*43*). A total of 0.5 μl of each DREADD virus was delivered in each site at 0.1 μl/min via an infusion pump. All viruses were obtained from Addgene. Exposed fiber ferrules and a protective black 3D-printed headcap were secured to the skull with dental cement. After surgery, rats were given cephalexin (15 mg/kg orally once a day) for 2 weeks to prevent any infection. Two rats in the CTRL group and one rat in the OFCi group lost their headcaps after surgery and were removed from the study.

### Fiber photometry

Dopamine-dependent fluorescence signals were recorded using custom-ordered multipronged fiber optic patch cables (200-μm diameter, 0.37 numerical aperture, Doric Lenses, Canada) that were attached to the optic fiber ferrules on the skull of the rats with brass sleeves (Thorlabs, NJ). Up to three fibers were connected at a time in each rat for recordings, and they were shielded and secured with a custom 3D-printed headcap-swivel shielding system that allowed for the relatively free movement of the rats without the use of optic commutators and prevented the spillover of light during recordings.

Recordings were conducted using an FP3002 system (Neurophotometrics, CA) by providing 470-nm (active signal) and 415-nm (isosbestic reference) excitation light through the patch cord in interleaved light-emitting diode pulses at 100 Hz (50-Hz acquisition rate for each channel). The light was reflected through a dichroic mirror and onto a 20× Olympus objective. Excitation power was measured at ~150 to 200 μW at the tip of the patch cord. Emitted fluorescent light was captured through the same cords, separated with an image splitting filter, and captured via a high-quantum-efficiency complementary metal-oxide semiconductor camera. Signals were acquired and synchronized with behavioral events using Bonsai (*41*). To avoid photobleaching, recordings were triggered so that the system was only active during behavioral trials (see the next section).

Signals were processed using custom scripts in MATLAB (MathWorks, MA). Raw fluorescence signals from the 470 (active) and 415 (reference) channels were first filtered with a fourth-order median filter. Then, the reference channel data were fitted to the active signal using a second-order polynomial fit, and the fitted data were subtracted from the active channel, removing dopamine-independent variations in fluorescence. The resulting signal was *z*-scored for each trial using the 10 s before each trial onset as a baseline. The resulting *z*-scores were normalized for each rat and session to the peak and baseline average, similar to previous work (*56*, *90*), to reduce between session variability.

For quality control, a group of rats (*n* = 2) was infused with GRAB-DA-mut, a mutated version of the GRAB-DA sensor that does not show dopamine-dependent fluorescence and is tested in similar tasks as the other experimental groups. All recordings in this group showed no fluorescence peaks around any behavior event, confirming that the signals we observed in the other groups were due to dopamine signaling (figs. S1 and S5).

### Behavioral apparatus and general procedures

Rats were trained and tested at least 8 weeks after the surgeries. All experiments were conducted in standard behavioral boxes (12″ × 10″ × 12″, Coulbourn Instruments, PA), which were individually housed in light- and sound-attenuating boxes (J. Garmon, JHU Psychology Machine Shop). Each box was equipped with a food cup (recessed magazine), a pellet dispenser, two wall speakers (one for white noise and the other for tones and sirens), a clicker, a house light, and a panel light. Auditory stimuli were played at 70 to 75 dB. Head entries into the food cup were measured on the basis of breaks of an infrared beam. For conditioning, 45 mg of sucrose pellets (5TUT, TestDiet, MO) was used as reinforcers. Intertrial intervals varied around ~6 min on average, and all cues lasted 10 s. Each rat experienced one session per day, and the order of presentation of each cue was counterbalanced across rats. Behavioral responses were quantified as the percentage of time that each rat spent in the food cup during the CS presentation.

A computer equipped with GS3 software (Coulbourn Instruments, PA) controlled the equipment. One Arduino microcontroller in each box recorded all events from the Coulbourn equipment using a strobe-based logic and relayed it as a serial input to the Bonsai photometry interface. One master Arduino relayed trial initiation and end from the Coulbourn interface to the FP3002 photometry hardware, triggering photometry recordings only during trials and turning them off during the intertrial intervals.

### Sensory preconditioning task

Rats were first lightly shaped to retrieve sucrose pellets from the food cup. They were then submitted to two sensory preconditioning sessions; these used a total of four different auditory stimuli, drawn from stock equipment available from Coulbourn, which included tone, siren, clicker, and white noise. Assignment of these stimuli to task cues, and the order in which they were presented during the session, was counterbalanced across rats. In each preconditioning session, rats received six blocked presentations of A-B pairs (A presented unexpectedly, followed by the immediate presentation of B) and C-D pairs (similar arrangement to the A-B pairs), with no direct reinforcement being present.

After preconditioning, rats in the main experiment were conditioned for six sessions, where they received randomized presentations of B immediately followed by the delivery of two sucrose pellets and D without any reinforcement, with six trials of each stimulus per session. In the final probe session, rats experience three reminder trials each where B was presented followed by reward, and D was presented with no outcome, immediately followed by blocked presentations, six each of A and C with no outcome. The ordering of cue presentation was counterbalanced. Before this probe, each rat received an intraperitoneal injection of JH60 (0.2 mg/kg, dissolved in 0.9% NaCl) and was left in their home cage for at least 20 min before the start of the session, to allow for the DREADD agonist to effectively inhibit transfected lOFC neurons in the OFCi group (*43*, *45*). Some rats in both groups did not show proper discriminative conditioning in both the conditioning and probe sessions. As the conditioning learning is required for revealing the latent learning that occurs in the preconditioning phase (*54*, *55*), only rats that responded twice as much to cue B than to cue D in the probe reminder trials were included in the final study, a criterion based on previous precondition work, which meant that four rats were excluded in the CTRL group and three rats in the OFCi group, with final *n* = 6 rats for CTRL and *n* = 8 rats for OFCi. Consistent with the interpretation that the weak conditioning might be a sign that these rats had general issues with learning, the dopamine signals in the NAcc and DMS in these rats (*n* = 7) during preconditioning was less reflective of prediction errors (fig. S8).

Two subsets of rats, infused only with GRAB-DA2m or GRAB-DA-mut in the NAcc and implanted with an optic fiber cannula in that same site, were subjected to the same aforementioned preconditioning protocol for two sessions, after which they underwent a third preconditioning session where, in the first three trials of each pairing, the cues were presented in the normal order (A → B and C → D), while in the subsequent three trials of each pairing, the cue order was switched (B → A and D → C). Another group of rats was infused only with GRAB-DA2m in the NAcc and then subjected to the standard preconditioning protocol for two sessions, after which they underwent a third preconditioning session where, in the first three trials of each pairing, the cues were presented in the normal order (A → B and C → D), and in the following three trials, the “predicted” cues (presented second) were swapped (A → D and C → B).

### Histological procedures

After completion of the experiment, rats were perfused with chilled phosphate-buffered saline (PBS) followed by 4% paraformaldehyde in PBS. The brains were then immersed in 18% sucrose in PBS for at least 24 hours and frozen. The brains were sliced at 40 μm, stained with DAPI (4′,6-diamidino-2-phenylindole; Vectashield-DAPI, Vector Lab, Burlingame, CA), and processed for immunohistochemical detection of green fluorescent protein (GFP; Fig. 1A and fig. S5, B and C). For immunohistochemistry, the brain slices were first blocked in 10% goat serum made in 0.1% Triton X-100/1× PBS and then incubated in anti-GFP antibodies (1/1000, room temperature, overnight, Mouse anti-GFP, 632381, Takara Bio USA, WI) followed by Alexa Fluor 488 secondary antibodies (1/200, room temperature, 2 hours, Donkey anti-mouse Alexa Fluor 488, 715-546-150, Jackson ImmunoResearch, PA). Fluorescence microscopy images of the slides were acquired with a BZ-X800 Keyence microscope (Fig. 1, A and B, and fig. S5, B and C).

### Statistical analyses

Statistical analyses were performed in GraphPad Prism (GraphPad Software, San Diego, CA). All analyses were performed using individual rats as the sampling unit, and error bars in figures denote the SEM. For planned comparisons between single measures across two groups, significance was tested with *t* tests if the data were normally distributed or Wilcoxon tests otherwise. For comparisons involving multiple factors, we used repeated-measures two-way and three-way ANOVAs combined with post hoc tests. Categorical differences were analyzed with chi-square tests. Correlations were tested with linear regression analysis or with robust nonlinear regressions using the ROUT regression method with *Q* = 1% (*91*), both with within-group tests of whether the slopes were significantly nonzero and between-group tests of slope differences. All tests were two-sided unless there was a strong prior hypothesis for a unidirectional effect. The statistical significance threshold for all tests was set at *P* < 0.05.
