# Supplementary material for: Striatal dopamine signals errors in prediction across different informational domains
Source: Sci Adv. 2025 Jul 9;11(28):eadq9684. doi: 10.1126/sciadv.adq9684 (PMC12239964; doi:10.1126/sciadv.adq9684)
Supplement: Supplementary file 1 — Supplementary Text Figs. S1 to S8 [file sciadv.adq9684_sm.pdf]

Science Advances
AAAS

Supplementary Materials for

Striatal dopamine signals errors in prediction across different informational domains

Kauê M. Costa *et al.*

Corresponding author: Kauê M. Costa, kaue.m.costa@gmail.com; Geoffrey Schoenbaum, geoffrey.schoenbaum@nih.gov

Sci. Adv. **11**, eadq9684 (2025)
DOI: 10.1126/sciadv.adq9684

This PDF file includes:

Supplementary Text
Figs. S1 to S8

Supplementary Text

Definition of terms and hypotheses

To avoid any misinterpretation of our findings, we should explicitly define the terms and constructs that we tested. We start by defining “attention”, “salience”, “novelty”, “value”, and “prediction error”. This is complicated, as most of these constructs are multi-dimensional (i.e., involve the integration and different variables) and interconnected. For the purposes of this study, “attention” to a given stimulus is defined as the alertness or the allocation of cognitive resources towards processing that stimulus, “salience” is defined as the ability of a stimulus to elicit attention, “novelty” is defined as the unfamiliarity of a subject with a given stimulus (i.e., how frequently it has observed this stimulus independently of any additional regularities in the sequence of observations), and “value” is defined as the subjective desire to experience a stimulus. “Prediction errors” are defined in their actual general sense as the difference between predicted (either based on memory or inference) and directly experienced stimuli. We believe these definitions follow the standard of the field. The *a priori* unifying factor across all these constructs is that they all influence and determine learning – the ability to acquire information about the external world.

These constructs are deeply intertwined. For example, attention is directly dependent on salience, which in turn may depend on several factors, like the intensity of a stimulus (e.g., the decibel intensity of an auditory stimulus, or the brightness of a light), its novelty, its value, and its predictedness, as well as the motivational state of the subject. The different components that comprise these constructs are dynamic and influence each other over time as a function of learning – for example, the salience of a cue determines the attention it receives and subsequently how quickly it can be associated with a valued outcome, but the pairing with a valued outcome feeds back and increases the cue’s salience over time. Novelty influences salience, and stimulus intensity itself also determines how quickly a subject learns about a novel stimulus. Across all these constructs, one common factor that influences them all is predictability, which is computed via the processing of prediction errors.

This is a critical point: all the tested constructs that influence dopamine responses in our experiments (and in the previous literature) depend on errors in predicting events across different domains of time and experience. This realization is what underlies our conclusion and proposal that the overarching computation being performed and conveyed by dopamine transmission is the comparison between predicted and currently experienced stimuli, whether they contain value or not.

Our sensory preconditioning experiment includes controls and additional steps to distinguish prediction errors from many different factors that may influence dopamine responses, including attention and salience. This is easier to understand if one considers the potential alternative results of our experiments if other factors were shaping dopamine responses. In Fig. S2 we compare the predictions for dopamine signal dynamics during sensory preconditioning (SPC) according to different competing hypotheses. Specifically:

1) If dopamine signals reflect only classic, model-free RPEs, then there should be no significant responses to neutral, value-less cues across the preconditioning procedure, since these cues do not hold value.
2) If dopamine signals reflect only sensory salience, then there should be equally sized responses to both cue types across the preconditioning procedure, since the sensory

properties of the cues – and thus their sensory salience - were unchanged. Note this is also true if dopamine reflects any static sensory features.

3) If dopamine signals reflect only novelty, then there should be equally large responses to both cues initially in the first phase of preconditioning that should diminish similarly to both cues over the course of training, since the relationship between the two cues would be of no import to their novelty.
4) If dopamine signals reflect only sensory prediction errors, namely violation of the predictive associations between the individual cues independently of their familiarity, then there should be equally large responses to both cues initially in the first phase of preconditioning, followed by a selective reduction to the predicted cues over the course of the first phase of preconditioning, with responses to unpredicted cues remaining equal or even increased.
5) Finally, if dopamine signals reflect a combination of sensory prediction errors and novelty (i.e., context-based predictions), then there should be equally large responses to both cues in early preconditioning and a gradual reduction of the responses over training, but with the responses to the predicted stimuli being reduced more than the responses to the unpredicted cues*, which is exactly what we observed and report in Fig. 2.*

Critically, any of these models would have been compatible with our previous causal manipulation experiment in SPC (*21*), as all that would be necessary, in principle, for those causal effects to be instantiated is an increased dopamine response to the neutral cues. Only a recording experiment like the one we conducted here can discriminate the different factors involved in shaping this learning-related signal, and only a model including a sensory prediction error can explain why the decline in signal is greater to the second cue in each pair.

The experiment reported in Fig. 3, where the order of presentation of A/B and C/D cue pairs was switched within a session, carried with it another set of predictions that would be categorically different depending on the variables at play. We performed it to confirm that the responses we observed in the original preconditioning task were indeed dependent on the predictive relationship between the cues, with the added value of replicating the main observed effect on a separate cohort and with a different dopamine sensor. The predictions for this experiment were:

1) If the greater reduction observed in the response to the second cue was caused only by a variable that imparted itself to the cue independently of its relational associations to the other cue (salience, attention, novelty, habituation, etc.), then we would expect that the responses to the previously predicted cues B and D would remain smaller than the responses to cues A and C.

2) If the observed reduction in response to the second cue was due to it being predicted by the first cue, then switching the order of presentation, and consequently the predictive contingency, should revert the response patterns, restoring the responses to B and D, which is precisely what we found and report in Fig. 3.

This experiment further ruled out a dominant effect of sensory salience *per se* in shaping these responses (as the cues were still the same, just in a different order). It also confirmed that the effect of predictability between the cues is additive to any effect of novelty (contextual associations) or habituation, as responses increased within session to the same stimuli that had

been previously experienced. This confirms that the differential suppression of responses to predicted cues was based on the relational structure of cue presentation.

Finally, in the experiment reported in Fig. 4, where the second cues in each pair were swapped, we tested whether the reduced dopamine responses to the cue that was presented second was indeed due to a specific stimulus-stimulus association or a general sensory habituation effect. The predictions were that if the swap did not change at all the responses to the cues that were presented second, then the suppression effect could be a form of more simple habituation. Conversely, if there was an increase in the dopamine response to the second cues after the swap, then this demonstrates that the prediction effect is cue-specific. Our results support the latter interpretation and show that the informational basis of the decline in dopamine to the second cue is specific. That is, that the change is not due to habituation over time to the presentation of a compound cue or due to general predictions that there is a second cue, but rather that this decline reflects the specific sensory content for the second cue that is predicted by the first in each pair.

Thus, considering these results overall, we found a pattern of dopamine responses across different experiments that is most compatible with dopamine responses to neutral cues being determined by sensory prediction errors and novelty (in context). This pattern of activity is formally incompatible with several other potential explanations, including the idea that "attentional" dopamine signals are shaped primarily by novelty and sensory salience independent of predictability. We also point out that novelty - the other main factor that affected the dopamine responses in the preconditioning phase - is also fundamentally computed as a prediction error between the experienced stimulus and all previously experienced stimuli retained in memory. Therefore, all the tested constructs that influence dopamine responses in our experiments (and in the previous literature) depend on errors in predicting events across different domains of time and experience.

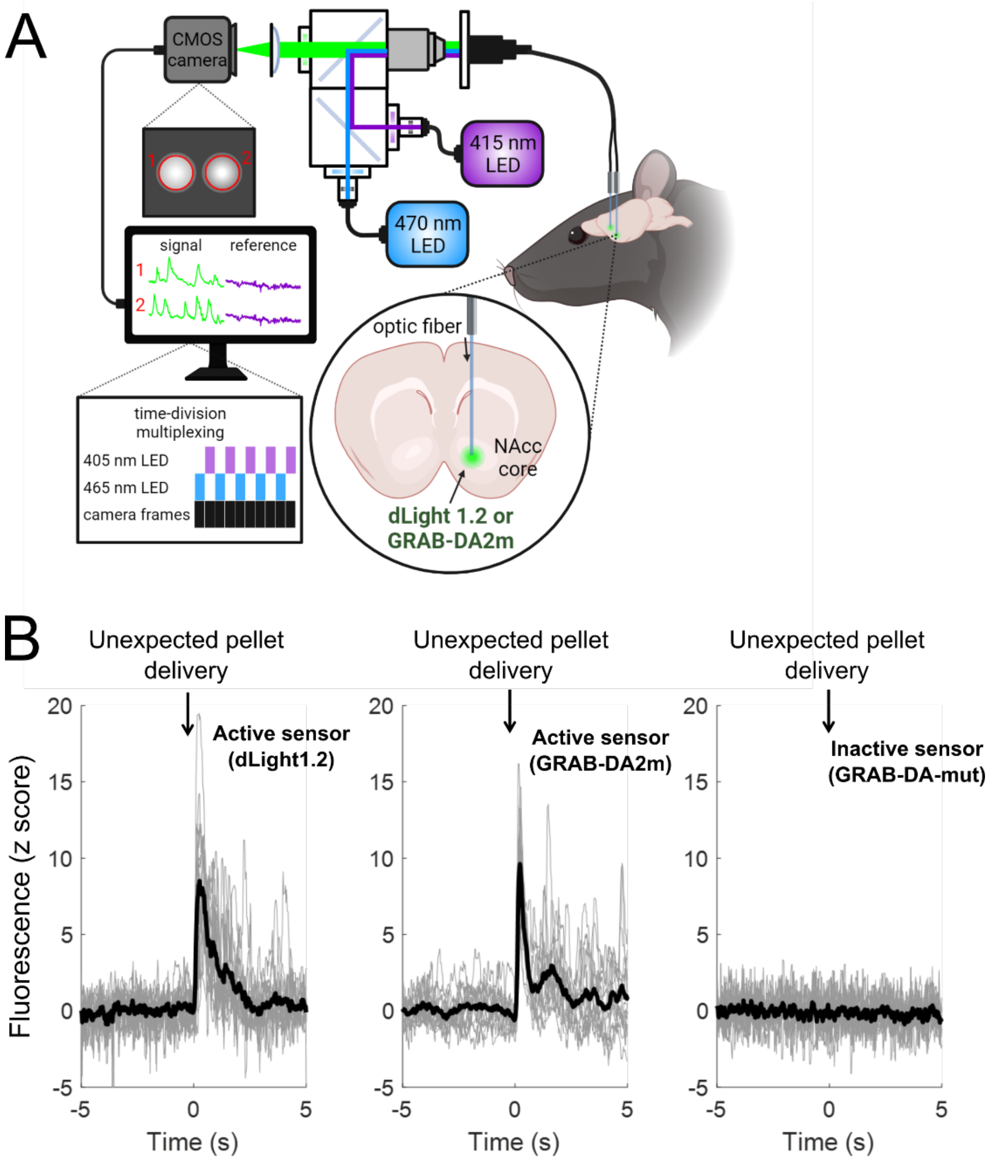

Fig. S1. Acquisition and validation of dopamine photometry recordings. (A): Cartoon schematic of the photometry approach for multi-site recording of dopamine signals in freely moving rats. Rats were implanted with multiple optic fiber cannulas and transfected with AAVs that led to the expression of dopamine sensors in target striatal regions, signals were collected using a Neurophotometrics FP3002 system, and reference (405 nm; ligand-insensitive fluorescence) and active (465) channels are separated using time-division multiplexing. Figure inspired by Martianova et al (*39*) and created with Biorender.com. **(B):** Validation of active dopamine sensors (dLight1.2 and GRAB-DA2m) by comparison with a mutated sensor that does not exhibit dopamine-dependent changes in fluorescence (*42*). The panels show data from representative rats, with bold black trace representing mean responses overlayed on gray traces representing 16 individual trials centered around unexpected pellet deliveries in the operant box. Note the clear dopamine responses in the rats transfected with the active sensors, but a complete absence of response in the rats transfected with the mutated control fluorophore.

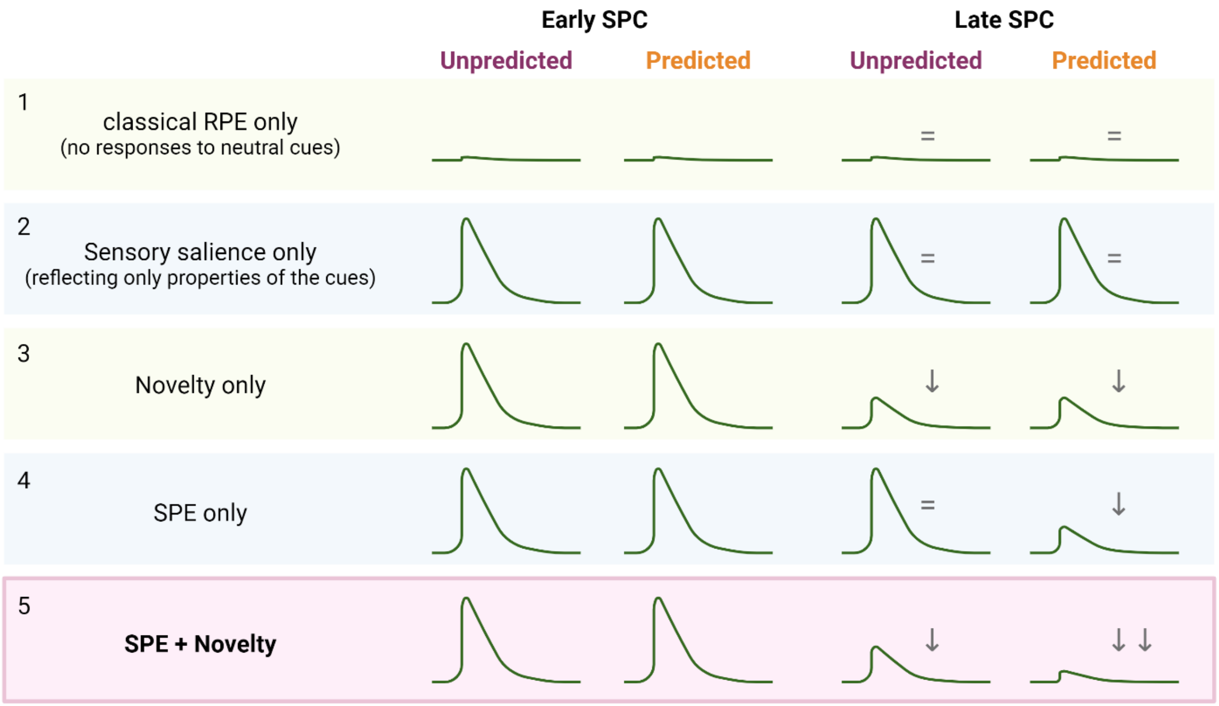

Fig. S2. Graphical representation of potential alternative patterns of dopamine responses during preconditioning. Here we consider potential alternatives to our findings, and which behavioral variables they would reflect had they been observed. 1) If dopamine signals reflect only classical, model-free RPEs, then there should be no significant responses to neutral, value-less cues across the whole preconditioning procedure. 2) If they reflect only sensory salience, then we should have observed equally sized responses to both cue types across the whole preconditioning procedure, since the sensory properties of the cues were unchanged. Note this is also true if they reflect any static sensory features. 3) If they reflect only novelty, then we would have expected large responses initially in the first phase of SPC, to both cues, which should diminish similarly over the course of training (as the relationship between the two cues would be of no importance). 4) If they reflect only sensory prediction errors, namely the predictive associations between the individual cues independently of their familiarity, then we would have seen equally large responses to both cue types initially in the first phase of preconditioning and a selective reduction in responses to the predicted cues in late SPC, with responses to unpredicted cues remaining equal or even increased. 5) Finally, if the responses reflect a combination of sensory prediction errors and novelty, then we would have expected to see equally large responses to both cue types in early preconditioning and a gradual reduction of the responses to both cue types over training, but with the responses to the predicted stimuli being reduced more than the responses to the unpredicted cues, which is exactly what we observed and report in Fig. 2.

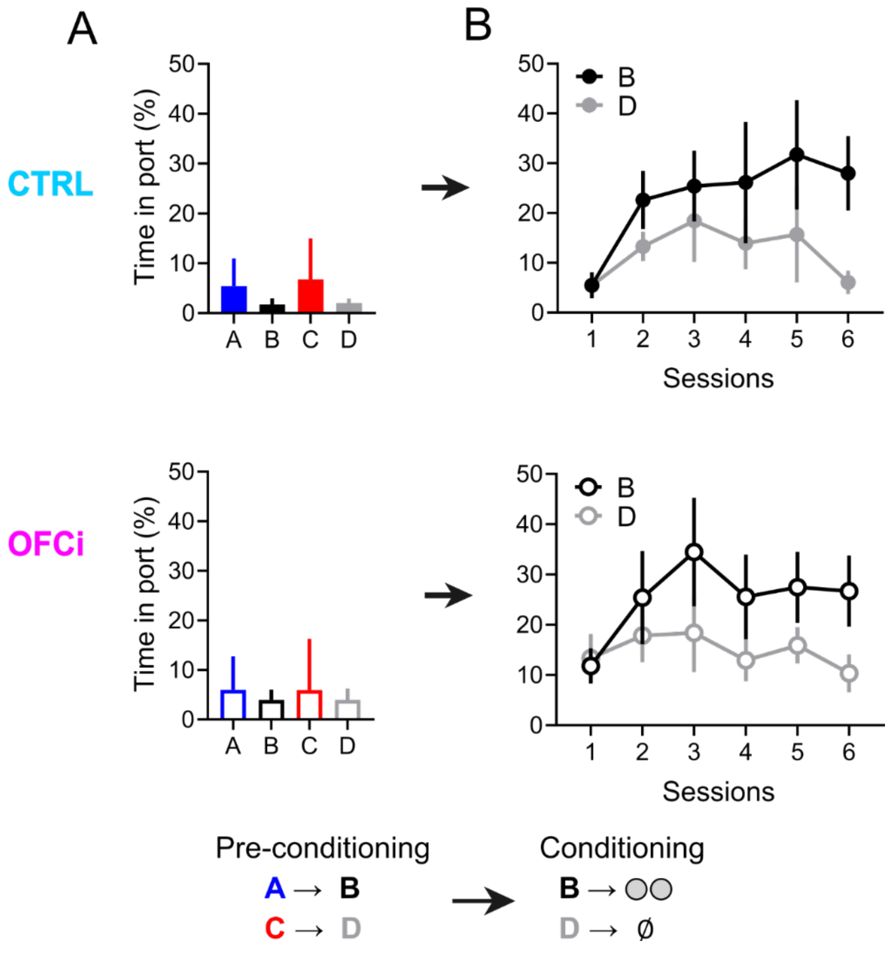

Fig. S3. Behavioral responses during preconditioning and conditioning, divided by groups. (A): Percentage of time in port during preconditioning for both CTRL and OFCi rats. **(B):** Same metric, but in conditioning. As mentioned in the main text, there was no difference in behavior performance between groups in these phases.

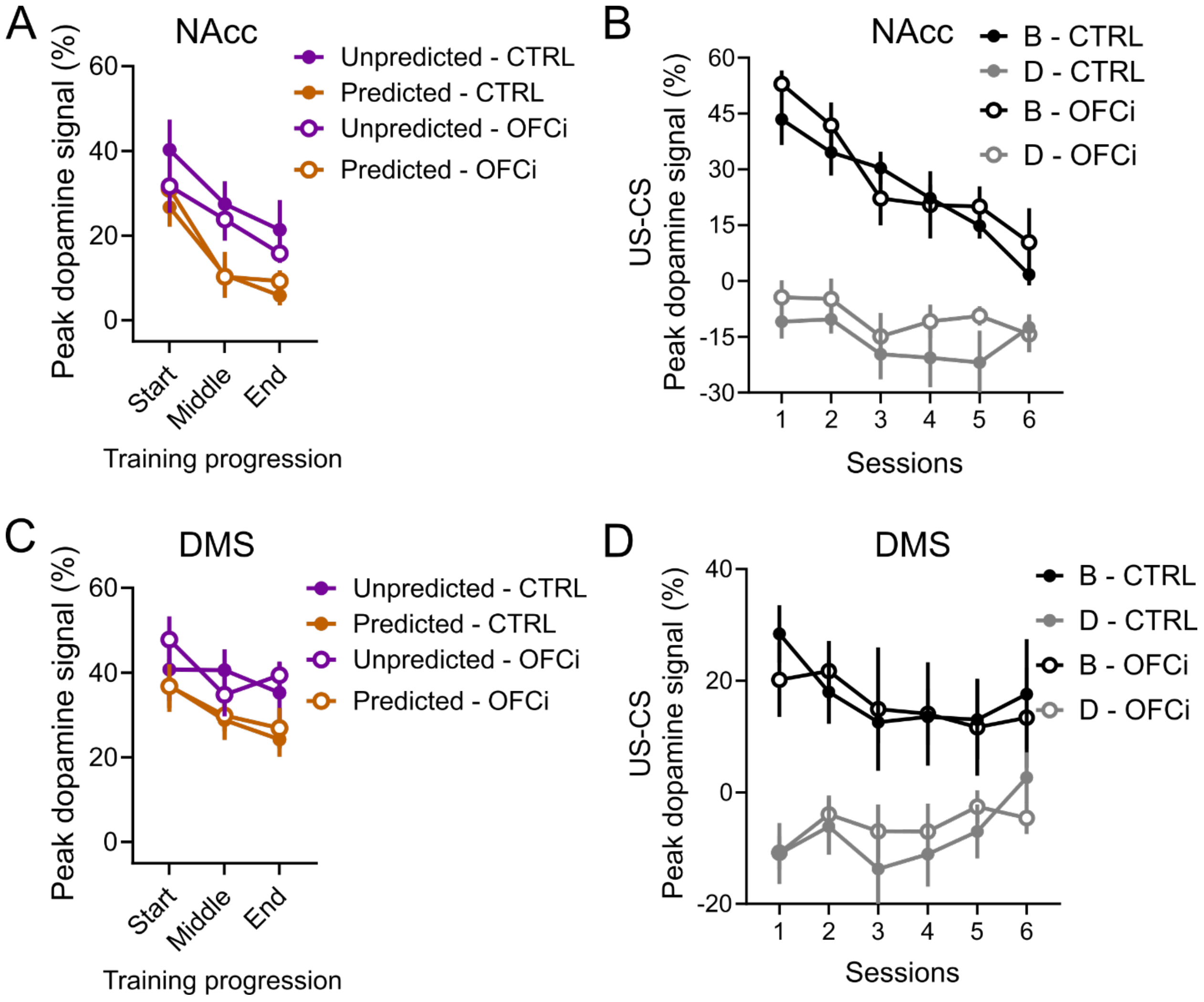

Fig. S4. Preconditioning and conditioning dopamine recordings, divided by groups. (A): Peak dopamine responses in the NAcc to predicted and unpredicted cues during preconditioning for both CTRL and OFCi rats. **(B):** Difference in peak NAcc dopamine responses to the US and CS – a measure of RPE representation – during conditioning sessions. **(C and D):** Same as A and B, but for the DMS recordings. As mentioned in the main text, there was no difference in quantified dopamine responses between groups in these phases (all group or group interaction effects had a $P>0.05$).

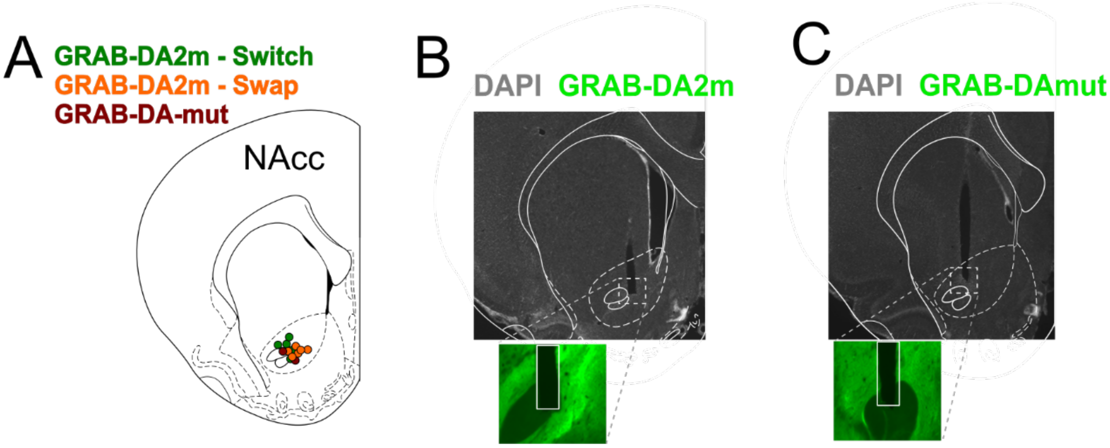

Fig. S5. Histological validation of rats transfected with GRAB-DA2m and GRAB-DA-mut. **(A):** Schematic representation of fiber tip locations in the brains of the five rats transfected with GRAB-DA2m and used for the preconditioning switch experiment (Switch, green), the six rats transfected with GRAB-DA2m and used for the preconditioning swap experiment (Swap, orange), and for the two rats transfected with GRAB-DA-mut, used as a control for our photometry recordings (dark red). **(B):** Representative histology of a rat transfected with GRAB-DA2m. **(C):** Same as B, but for a rat transfected with GRAB-DA-mut.

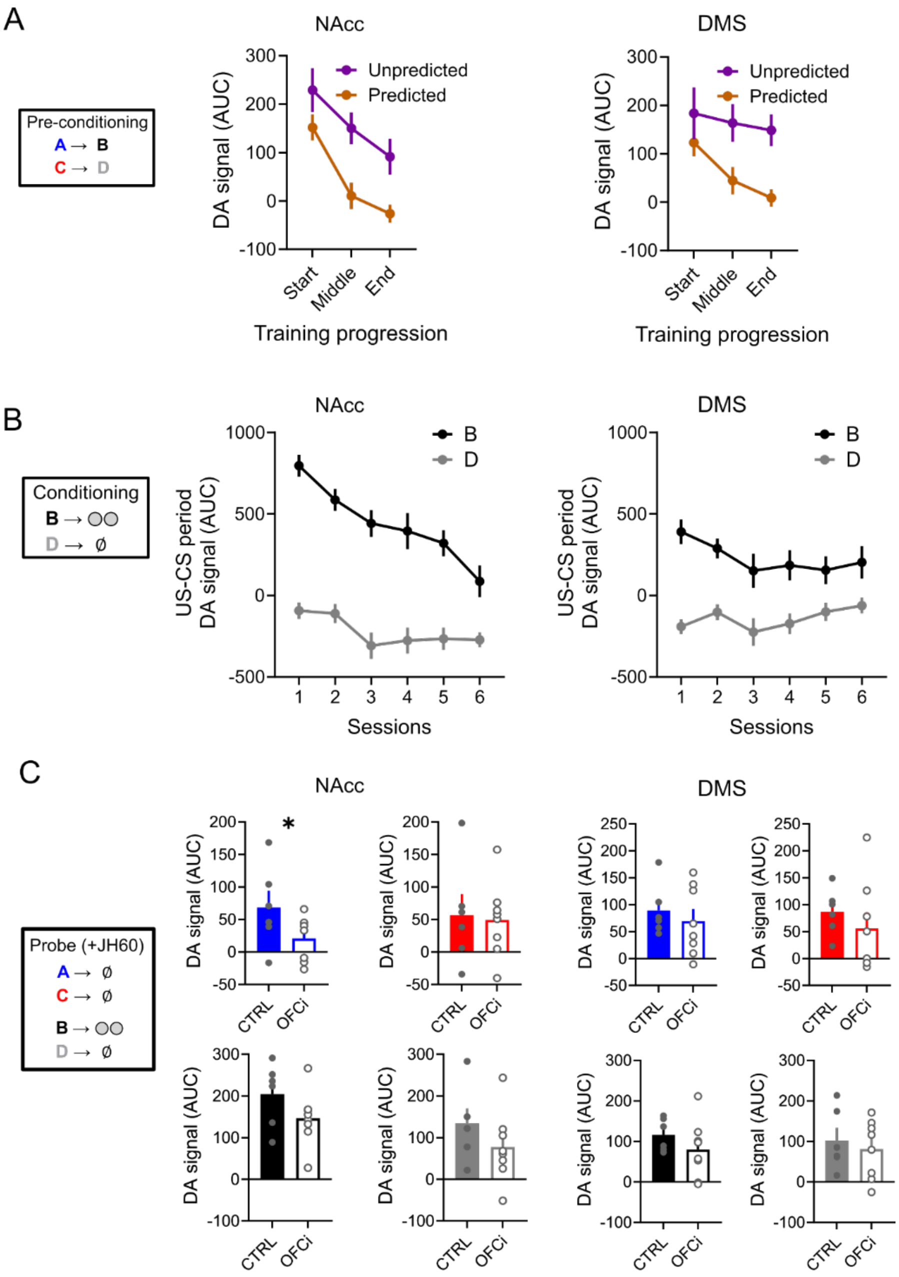

Fig. S6. Analysis of area under the curve (AUC) of dopamine signals. **(A)**: AUC values for dopamine responses during the SPC phase of the main experiment. 2-way ANOVA revealed significant differences in both the NAcc (trial progression: *P*<0.0001****, $F_{(2,54)}$ = 16.41; cue predictability: *P*=0.0005***, $F_{(1,27)}$ = 15.54) and the DMS (trial progression: *P*=0.0349*, $F_{(2,54)}$ = 3.572; cue predictability: *P*=0.0009***, $F_{(1,27)}$ = 13.80) that matched the pattern for the peak analysis showed in Fig. 2. **(B):** AUC values for dopamine responses during the conditioning phase of the main experiment. 2-way ANOVA revealed significant differences in both the NAcc (session progression: *P*<0.0001****, $F_{(5,65)}$ = 12.41; cues: *P*<0.0001****, $F_{(1,13)}$ = 184.6; interaction: *P*=0.0001***, $F_{(5,65)}$ = 5.915) and the DMS (cues: *P*<0.0001****, $F_{(1,13)}$ = 35.89; interaction: *P*=0.0086**, $F_{(5,65)}$ = 3.402) that matched the pattern for the peak analysis showed in Fig. 5. **(C):** AUC values for dopamine responses during the probe phase of the main experiment. Unpaired t-tests revealed a significant difference between CTRL and OFCi group in responses to cue A (one tailed unpaired t-test, *P*=0.045*), in line with the stronger effects observed in analyses of peak responses presented in Fig. 6.

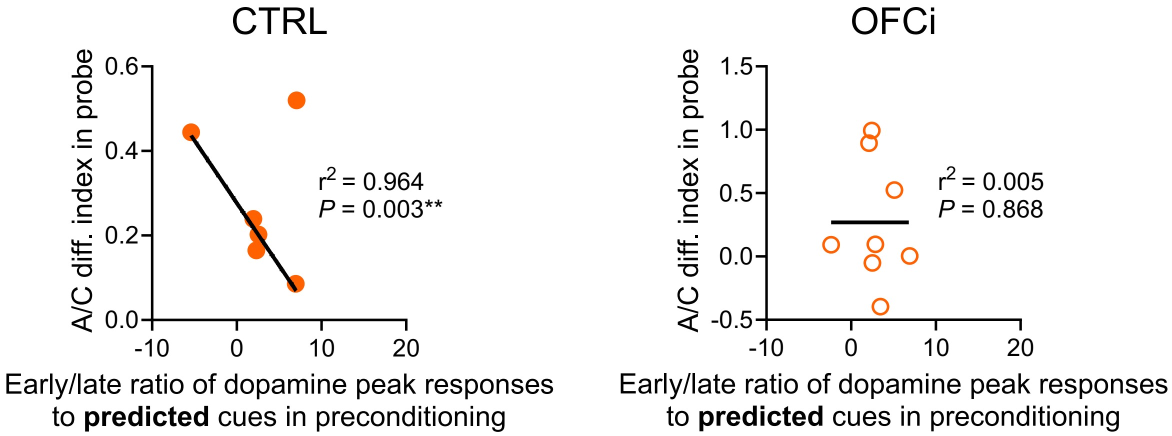

Fig. S7. NAcc dopamine SPE signaling in preconditioning predict behavior in the probe session. Panels represent the relationship between the ratio of NAcc dopamine peak responses to cues, between the early and late phases of preconditioning (peaks in the first two trials divided by peaks in the last two trials, or the degree to which a given cue response was suppressed by preconditioning training), and the A/C difference index in the probe (a normalized measure of the behavioral sensory preconditioning effect). Regression lines and text are the results of regression analysis (ROUT regression with Q=1%) (*95*). The suppression of dopamine responses to predicted cues (B and D), from start to end of preconditioning, in the CTRL group, was inversely correlated with the magnitude of A/C discrimination during the probe. Importantly, this relationship is not observed in the OFCi group. $^{**}P<0.01$.

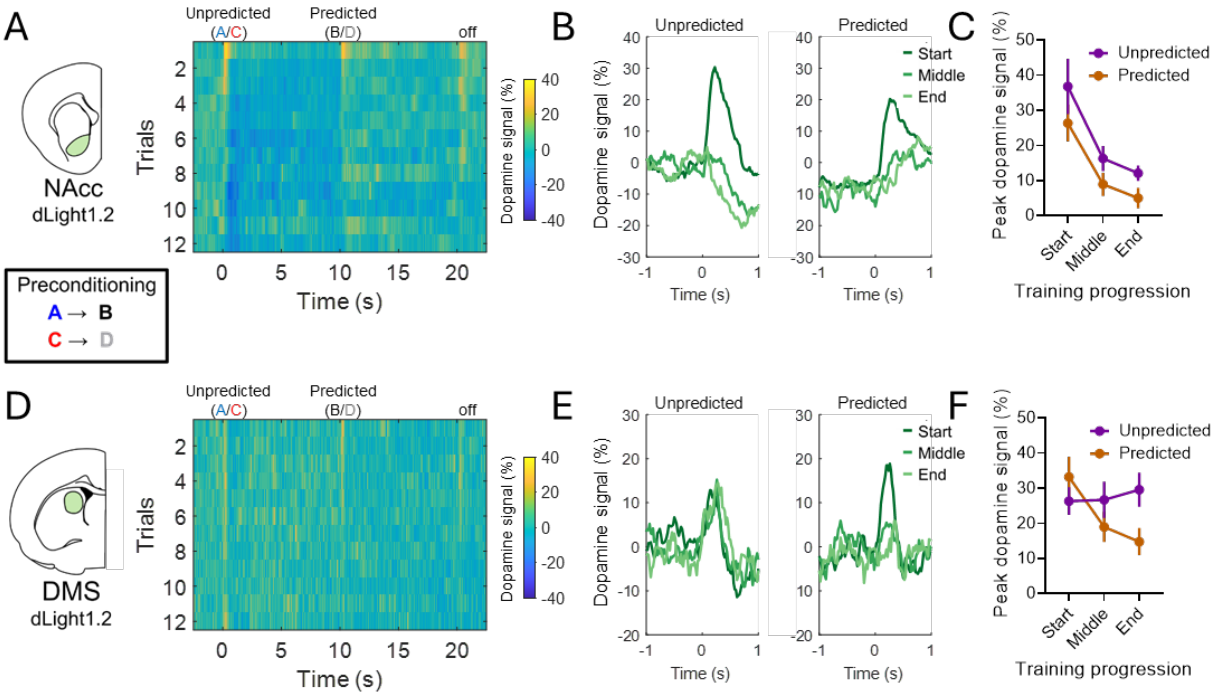

Fig. S8. Dopamine signals in NAcc and DMS during preconditioning from rats that showed weak conditioning and were removed from later analysis. (A): Heatmap representation of average NAcc dopamine responses in the preconditioning phase, during all 12 preconditioning trials, recorded with dLight1.2, from excluded rats that did not show strong conditioning discrimination (N= 7 rats). Note that unlike for the rats that showed good conditioning responses, in these rats NAcc dopamine responses to predicted and unpredicted cues were not significantly different (2-way ANOVA for effect of trial progression and cue predictability; trial effect: P=0.0001***, $F_{(2,12)} = 20.9$; cue predictability effects: P>0.05, $F_{(1,6)} = 5.01$; interaction effect: P>0.05, $F_{(2,12)} = 0.09$). Indeed, positive NAcc dopamine responses to all cues seemed to be all but eliminated within the first few trials, perhaps suggesting that these rats were less engaged in the task, although this is speculation. **(B):** Average traces of NAcc dopamine responses to predicted and unpredicted cues during the start (first two trials), middle (trials 6 and 7), and end (trials 11 and 12) of the preconditioning phase for the same "bad learner" rats. **(C):** Peak NAcc dopamine responses measured over the course of the preconditioning training. **(D-F):** similar to A-C, but for DMS dopamine signals, simultaneously recorded with NAcc signals. In the DMS of these rats, the differential reduction of dopamine responses to predicted and unpredicted cues was still significant, as there was an interaction effect between cue and trial progression (2-way ANOVA for effect of trial progression and cue predictability; trial effect: P>0.05, $F_{(2,12)} = 2.57$; cue predictability effects: P>0.05, $F_{(1,6)} = 1.302$; interaction effect: P=0.005**, $F_{(2,12)} = 8.443$). Data are represented as mean ± SEM. **P<0.01; ***P<0.001.
